# A systematic review and activation likelihood estimation meta-analysis of the central innervation of the lower urinary tract: Pelvic floor motor control and micturition

**DOI:** 10.1371/journal.pone.0246042

**Published:** 2021-02-03

**Authors:** Ilse M. Groenendijk, Ulrich Mehnert, Jan Groen, Becky D. Clarkson, Jeroen R. Scheepe, Bertil F. M. Blok

**Affiliations:** 1 Department of Urology, Erasmus Medical Center, Erasmus University, Rotterdam, The Netherlands; 2 Department of Neuro-Urology, Balgrist University Hospital, University of Zürich, Zürich, Switzerland; 3 Division of Geriatric Medicine, University of Pittsburgh, Pittsburgh, PA, United States of America; University Medical Center Utrecht, NETHERLANDS

## Abstract

**Purpose:**

Functional neuroimaging is a powerful and versatile tool to investigate central lower urinary tract (LUT) control. Despite the increasing body of literature there is a lack of comprehensive overviews on LUT control. Thus, we aimed to execute a coordinate based meta-analysis of all PET and fMRI evidence on descending central LUT control, i.e. pelvic floor muscle contraction (PFMC) and micturition.

**Materials and methods:**

A systematic literature search of all relevant libraries was performed in August 2020. Coordinates of activity were extracted from eligible studies to perform an activation likelihood estimation (ALE) using a threshold of uncorrected p <0.001.

**Results:**

20 of 6858 identified studies, published between 1997 and 2020, were included. Twelve studies investigated PFMC (1xPET, 11xfMRI) and eight micturition (3xPET, 5xfMRI). The PFMC ALE analysis (n = 181, 133 foci) showed clusters in the primary motor cortex, supplementary motor cortex, cingulate gyrus, frontal gyrus, thalamus, supramarginal gyrus, and cerebellum. The micturition ALE analysis (n = 107, 98 foci) showed active clusters in the dorsal pons, including the pontine micturition center, the periaqueductal gray, cingulate gyrus, frontal gyrus, insula and ventral pons. Overlap of PFMC and micturition was found in the cingulate gyrus and thalamus.

**Conclusions:**

For the first time the involved core brain areas of LUT motor control were determined using ALE. Furthermore, the involved brain areas for PFMC and micturition are partially distinct. Further neuroimaging studies are required to extend this ALE analysis and determine the differences between a healthy and a dysfunctional LUT. This requires standardization of protocols and task-execution.

## Introduction

The neuronal control of the lower urinary tract is based on multilevel circuits, i.e. peripheral nerves, autonomic ganglia, spinal cord pathways, and supraspinal centers [1]. The latter allow for the voluntary control and proper coordination of LUT function including synergic micturition (that is, bladder neck and external urethral sphincter relaxation during detrusor contraction) [2–5].

Functional neuroimaging is a powerful and versatile tool to investigate the neural structures and processes involved in central lower urinary tract (LUT) control. The LUT motor control is usually only perceived as storage and micturition (detrusor control as autonomous activity), but the descending LUT control also includes voluntary, conscious pelvic floor muscle contractions (PFMC). Voluntary PFMC are a proxy for, but not the same as, the involuntary tonic contraction employed during the storage phase. Especially since PFMC tends to be a voluntary “backup” mechanism which is employed during very strong urge to void or defecate. Despite the increasing body of literature, publications are scattered among various medical disciplines and there is a lack of comprehensive overview on the descending central LUT control [2–12]. Hence, it would be of great interest to get a structured overview on this topic and determine the most relevant brain areas involved in LUT motor control. This is of importance for a better understanding of the composition of supraspinal LUT control networks and to be able to distinguish between networks involved in healthy control as opposed to LUT dysfunction. This would contribute to elucidate the pathophysiology of some highly prevalent diseases within functional urology, like overactive and underactive bladder (OAB/UAB), bladder pain syndrome (BPS) and dysfunctional voiding.

A well-established approach to conduct a coordinate-based meta-analysis (CBMA) of the existing neuroimaging data in order to achieve a comprehensive overview of the relevant brain regions involved in functional tasks of interest is Activation Likelihood Estimation (ALE) analysis [13]. ALE analyses determine the statistical probability of brain regions being consistently activated during a specific task.

The aim of the present systematic review is to summarize the existing evidence on the supraspinal motor control of the LUT in humans, i.e. micturition and PFMC, and to determine in an exploratory fashion the core brain areas involved in these functions using ALE.

## Materials and methods

### Study registration

This systematic review was conducted according to the Preferred Reporting Items for Systematic Reviews and Meta-Analysis (PRISMA) statement. The study protocol was registered on PROSPERO (CRD42016047488 https://www.crd.york.ac.uk/PROSPERO/).

### Literature search

A systematic search of all relevant publications was conducted in PubMed, EMBASE, Medline, Scopus, Web of Science, and the Cochrane library. A search was conducted including all publications until August 2020, S1 File contains the used search terms. Manual reference checks of accepted papers in recent reviews and included papers were performed as supplement to the electronic search.

### Eligibility criteria

All original publications on neuroimaging of lower urinary tract control in humans were eligible for full-text retrieval.

The use of a comprehensive search strategy resulted in a highly heterogeneous body of data which included a variety of neuroimaging techniques, populations, settings and protocols. In order to optimally utilize this extensive literature search, it was decided to split the extracted studies into smaller components addressing more concise research questions to get more precise answers. The current review focused on the assessment of LUT motor control using PET and fMRI. Therefore literature concerning PFMC or micturition using other neuroimaging techniques like diffusion MRI (tractography), DTI, NIRS, SPECT was excluded.

Hence, inclusion criteria were studies using fMRI or PET when performing micturition or pelvic floor muscle contractions that described the coordinates (in stereotactic space i.e. Talaraich (TAL) or MNI) of active clusters found during the performed task. Duplicates, abstracts only, conference proceedings, non-English text publications, non-human research and reviews were excluded.

### Selection of studies

Titles and abstracts were screened in Endnote (EndNote X9; Thomson Reuters, Philadelphia, PA, USA) by U.M. and B.B. and discrepancies were resolved by I.G. The selected articles were full text screened for eligibility by J.G. and I.G. using a standardized screening form, and discrepancies were discussed and resolved by a third reviewer (B.B.).

### Data extraction

The data were independently extracted from the included full-text publications by two reviewers (J.G. and I.G.) using a standardized form. Any discrepancies were discussed and resolved by the third reviewer (B.B.). Data extracted were: general descriptive information of the studies, sample sizes, study population, characteristics describing the scanning protocol, applied analysis and the reported coordinates of supraspinal activity (raw data).

### Raw data

The extracted raw data were the coordinates of the activated clusters described by the included studies. The coordinates are shown in the orientation in which they were originally described, i.e. MNI or TAL. Most included studies focusing on micturition, separately reported the coordinates of the patients with successful micturition and unsuccessful micturition. The current study only extracted the coordinates of the subjects with successful micturition if the data was reported separately. In two studies the distinction between successful and unsuccessful micturition was not made.

### Primary outcome: Activation Likelihood Estimation (ALE)

Coordinate-based meta-analyses of neuroimaging results were performed using GingerALE software (version 2.3.6) available on the BrainMap website (http://brainmap.org/software.html). ALE analysis uses all the reported foci from the included studies as a spatial probability distribution centered at the given coordinates. The analysis accommodates the spatial uncertainty of neuroimaging findings and uses a spatial variance model. Finally, the convergence of foci is tested against the null-hypothesis of random distribution of foci, creating the ALE score for each cluster. The TAL coordinates were converted to MNI space using the icbm2tal transform within the GingerALE software [14]. A lenient threshold was used, taking the amount of data subjects into account (uncorrected p <0.001, minimal Volume of 100 mm^3^). Results were presented on an MNI template using Mango, multi-image viewing software (http://ric.uthscsa.edu/mango/).

### Risk of bias assessment

The Cochrane Risk of Bias Assessment tool together with an assessment of the main confounders following recommendations of the Cochrane handbook for nonrandomized comparative studies [15,16] were used to perform a risk of bias analysis for included nonrandomized comparative studies. Firstly, a manual was developed for scoring the added confounders. Secondly, the main and added confounders were independently scored by two authors (J.G. and I.G.) and discrepancies were discussed. Table 1 shows the used confounders and how they were scored.

**Table 1:** Scoring manual for the confounders in the risk of bias assessment.

Confounders	If yes: low risk If doubtful: unclear risk If not: high risk In case of multiple items in 1 confounder: 1 missing: unclear risk >1 missing: high risk
A priori protocol	Did the authors use and describe a clear protocol?
Age	Did the authors report the age of patients? Is there were different groups to compare, were they age matched?
Gender	Did the authors report the gender of patients? If there were different groups to compare, were they gender matched?
Patient selection	Did the authors clearly state in- and exclusion criteria? Which LUT symptoms and/or dysfunctions had included patients? Were handedness and concomitant medications reported?
Task and scan paradigm	Did the authors report on the task they investigated and how this task was applied (mode of bladder filling, repetition of task, control task, scanning sequence)?
Recording details and scan parameters/settings	Did the authors report all parameters/settings used for the recordings of supraspinal signals? (fMRI: TR, TE, FoV, ST) (PET: camera position, GBq, start time after injection, repeat injection, voxel size)
Data analysis	Did the authors report on data analysis (software, version, analysis steps, smoothing)?
Results	Did the authors report on all result details (coordinated in MNI or Talairach, cluster size of supraspinal areas, z-score, significance lever, error correction)?

## Results

**Fig 1:**
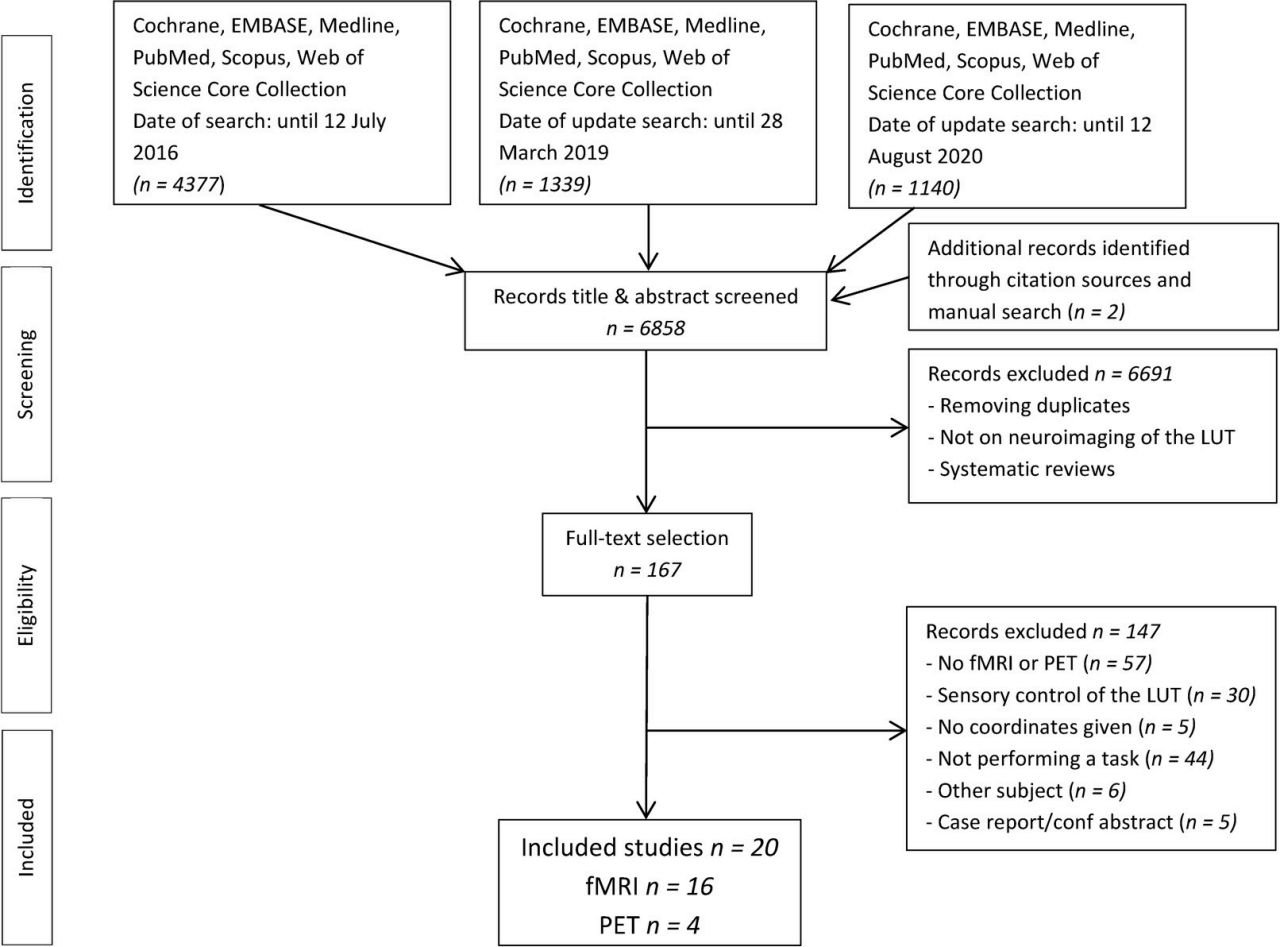
The PRISMA flow diagram.

The PRISMA flow diagram in Fig 1 shows the results of the literature search and the study selection. The initially conducted search resulted in 6858 articles. After removing duplicates and title and abstract screening, 167 articles remained for full-text selection and in total 20 studies were included in this systematic review.

### Study characteristics

Tables 2 and 3 show the study characteristics of the included studies using fMRI and PET, respectively. The following information was included in Tables 2 and 3: number of subjects, age, gender, performed task, concomitant/control task, task repetition, bladder status, way of bladder filling, scan details and analysis details (smoothing, MNI/TAL, threshold).

**Table 2:** Study characteristics of fMRI studies.

Main study details					Task paradigm							Scan details and analysis						
Study	N	Study population	Age	M/F	Main task	Concomitant tasks	Control task	Design: Block or event related	Main task repetition	Bladder status	Bladder filling	Tesla	-TR -TE -FoV -ST	Software	Smooth Gaussian	Group analyses model	MNI or TAL	Threshold P value
**PELVIC FLOOR MUSCLE CONTRACTION**																		
																		
Groenendijk, I.M. et al (2020) [17]	13	Healthy volunteers	29.6	13/0	PFMC	None	Tongue movement	Block	12	Empty	-	7T	- 2000 - 25 - 223 - 1.75	SPM8	2.5 mm	Random effects	MNI	Uncorrected <0.001
Seseke, S. et al (2019) [18]	11	Multiple sclerosis	54.5	3/8	PFMC	None	None	Block	15	Full	Natural	3T	-2250 -3.26 - -3	Brain voyager QX	5 mm	Random effects	TAL	Cluster based cor <0.05
Kutch, J.J et al (2015) [19]	14	Healthy volunteers	-	14/0	PFMC	None	Right hand contraction	Block	6	Empty	-	3T	- 2500 - 34.5 - 220 - 3	FMRIB	5 mm	Mixed effects	MNI	Cluster based cor <0.05
Krhut, J. et al (2014) [20]	23	Healthy volunteers	20–68	0/23	PFMC	None	None	Block	10	Unknown	-	3T	- 2000 - 20 - 192 -	SPM8	6 mm	Random effects	MNI	Uncorrected P = 0.0001
Schrum, A. et al (2011) [21]	17	Healthy volunteers	28.9	17/0	PFMC	None	Toe movement	Block	6 fast 6 slow	Empty	-	3T	- 2500 - 34.5 - 224 - 3	SPM5	6 mm	Random effects	MNI	FWE corrected <0.05
Seseke, S. et al (2008) [22]	12	Healthy volunteers	32.4	12/0	PFMC	None	Rest	Block	15	Full	Natural	3T	- 2000 - 36 - - 4	Brain voyager QX	8 mm	Random effects	TAL	FDR <0.05
Kuhtz-Buschbeck, J.P. et al (2007) [7]	30	Healthy volunteers	26.6	15/15	PFMC	None	Rest	Block	5	Variable within subjects	-	1.5T	- - 50 - 220 - 3	SPM2	7 mm	Random effects	MNI	FEW p = variable
Seseke, S. (2006) [23]	11	Healthy volunteers	30	0/11	PFMC	None	Rest	Block	15	Full	Natural	3T	- 2000 - 36 - - 4	Brain voyager QX	4 mm	Random effects	TAL	FDR <0.05
Di Gangi Herms, A.M.R. et al (2006) [24]	10	Stress urinary incontinence	57	0/10	PFMC	Pressure balloon in vagina	Fist clenching	Block	11	Empty	-	3T	- 3000 - 30 - - 3	SPM2	9 mm	-	MNI	Corrected <0.05
Kuhtz-Buschbeck, J.P. et al (2005) [25]	22	Healthy volunteers	24.5	0/22	PFMC	Initiate micturition, but not void	Rest	Block	5	Full (±300cc)	Natural	1.5T	- - 50 - 220 - 3	SPM2	7 mm	Random effects	MNI	Corrected p = variable
Zhang, H. et al (2005) [10]	12	Healthy volunteers	23.8	12/0	PFMC	None	PFMC with empty bladder	Block	8	Full vs empty	Natural	3T	- 2900 - 30 - 230 - 5	SPM99	8 mm	Conjunction group analysis	TAL	Corrected P = variable
**MICTURITION**																		
Khavari, R. et a; (2017) [26]	16^a^	Multiple sclerosis	46.8	0/16	Initiated micturition	None	Healthy controls	Event related	4	Full	Catheter	3T	- 3000 - 35 - 240 - 4	AFNI	5 mm	-	MNI	P = 0.05
Michels, L. et al (2015) [5]	14^b^	Healthy volunteers	26.4	14/0	Micturition	None	Rest	Event related	10	Full	Natural	3T	- 3000 - 35 - 220 - 3	SPM5	8 mm	Random effects	MNI	FDR P = 0.001
Shy, M. et al (2014) [27]	10	Healthy volunteers	32.4	0/10	Micturition	None	Rest	Event related	4	Full	Catheter	3T	- 3000 - - - 4	AFNI	-	-	MNI	Uncorrected P = 0.00027
Krhut, J. et al (2012) [28]	6^c^	Healthy volunteers	49.6	0/6	Micturition	None	None	Event related	3	Full	Catheter	3T	- 3000 - 30 - - 3	SPM5	8 mm	-	MNI	Uncorrected P = 0.02
Kuhtz-Buschbeck, J.P et al (2009) [4]	33	Healthy volunteers	26.4	16/17	Initiated micturition, but not void	None	Different bladder volumes	Block	5	Full	Natural	1.5T	- - 50 - 220 - 3	SPM2		Random effects	MNI	FEW P = variable

Abbreviations: TR: Repetition time, TE: Time of echo, FoV: Field of View, St: Slice thickness, TAL: Talairach, PFMC: Pelvic floor muscle contraction. a Of 16 patients, 7 performed successful micturition but data was not reported separately. b Originally 22 patients were included but only 14 performed successful micturition and data was reported separately c Originally 12 patients were included but only 6 performed successful micturition and data was reported separately.

**Table 3:** Study characteristics of PET studies.

Main study details						Task paradigm						Scan details and analysis							
Study	N	study population	Age	M/F	Main task	Concomitant tasks	Control task	Main task repetition	Design: Block or event related	Bladder status	Bladder filling	Camera position-below-above	- GBq -start scan after injection - data acquisition - repeat injection - voxel size	Software	Smooth Gaussian	Group analyses model	MNI or TAL	Threshold P value	
**PELVIC FLOOR MUSCLE CONTRACTION**																			
Blok B.F.M et al (1997) [2]	6	Healthy volunteers	21–24	0/6	PFMC	None	rest	1	Block	empty	-	- -20 - 76	- 1.85 -23 sec - 90 sec -4 -2.2	SPM96	12	ANCOVA	Tal		Uncorrected <0.001
**MICTURITION**																			
Nour S. et al (2000) [29]	8^a^	Healthy volunteers	23.4 ± 1.1 (22–25)	8/0	Micturition	None	rest	2–4	Event related	filled to desire	Catheter	- -	- 0.4 - 30 sec - 90 sec - 10–12 -	SPM 96	10	-	Tal		variable
Blok, B.F.M et al (1998) [6]	10^b^	Healthy volunteers	27 (20–51)	0/10	Micturition	None	Empty bladder	1	Block	full	Natural	- 28 mm - 48 mm	- 1.85 - 23 sec - 90 sec - 4 - 2.2	SPM95	8	ANCOVA	Tal		Uncorrected <0.001
Blok B.F.M et al (1997) [3]	10^c^	Healthy volunteers	32.3 (21–50)	10/0	Micturition	None	Empty bladder	1	Block	full	Natural	-28 mm -44 mm	- 1.85 - 23 sec - 90 sec - 4 - 2.2	SPM 95	8	ANCOVA	Tal		Uncorrected P = variable

Abbreviations: GBq: The amount of gigabecquerel H215O (in case of Blok et al diluted in saline) injected before PET scan. TAL: Talairach. a 12 patients were included but only 8 performed successful micturition b 18 patients were included but only 10 performed successful micturition c 17 patients were included but only 10 performed successful micturition.

### Raw data

#### Pelvic floor muscle contraction

Twelve studies investigated active brain areas during PFMC. One of them [3] did this using PET, the others all used fMRI.

Of these 12 studies, 9 found active clusters in the primary motor cortex (M1), either in the right or left hemicortex, or both. Seven studies found activation in the supplementary motor area (SMA). Some studies reported activation in the frontal lobe, with coordinates widespread through the frontal lobe. The putamen showed active clusters in 4 studies, the thalamus and the insula both showed active clusters in 6 studies. Active clusters in the cerebellum were described in 9 studies. S1 Table shows the coordinates of all brain areas that have been consistently found in at least 3 of the included studies. If, in a single study, more than one cluster was identified in the same brain area, the cluster with the highest T- or Z-value is displayed in S1 Table. All active clusters described in individual studies only, are summarized in S2 Table.

#### Micturition

Eight studies investigated brain areas involved in micturition, of which 5 used fMRI and 3 used PET. The study of Kuhtz-Buschbeck et al. 2009 [4], investigated initiation of voiding only, i.e. participants prevented voiding by contracting the pelvic floor when micturition was about to start, all other studies investigated real micturition. Only in the study of Khavari et al. data of patients with successful micturition (n = 7) and unsuccessful micturition (n = 9) were not reported separately. In four studies, patients could sign (hand or vocally) when they were about to start micturition [26–29]. Two studies used a flow/urine detector to detect the start of micturition [5,6] and in two studies patients were instructed when to start micturition [2,4]. Six studies found activation in the periaqueductal gray (PAG) and 5 in the dorsal pons, including pontine micturition center (PMC). Cingulate gyrus was active in 6 studies. The insula, the thalamus, the mid frontal gyrus and the cerebellum were activated in 4 studies. The inferior frontal gyrus (Brodmann area 11, 44–47) was found active in all studies. S3 Table shows peak coordinates of brain areas that were reported to be active in at least 3 of the included studies. If, in a single study, more than one cluster was found in the same brain area, the cluster with the highest T- or Z-value is displayed in S3 Table. All other active clusters described in individual studies only, are summarized in S4 Table.

### Primary outcome: Activation likelihood estimation

#### Pelvic floor muscle contraction

**Fig 2:**
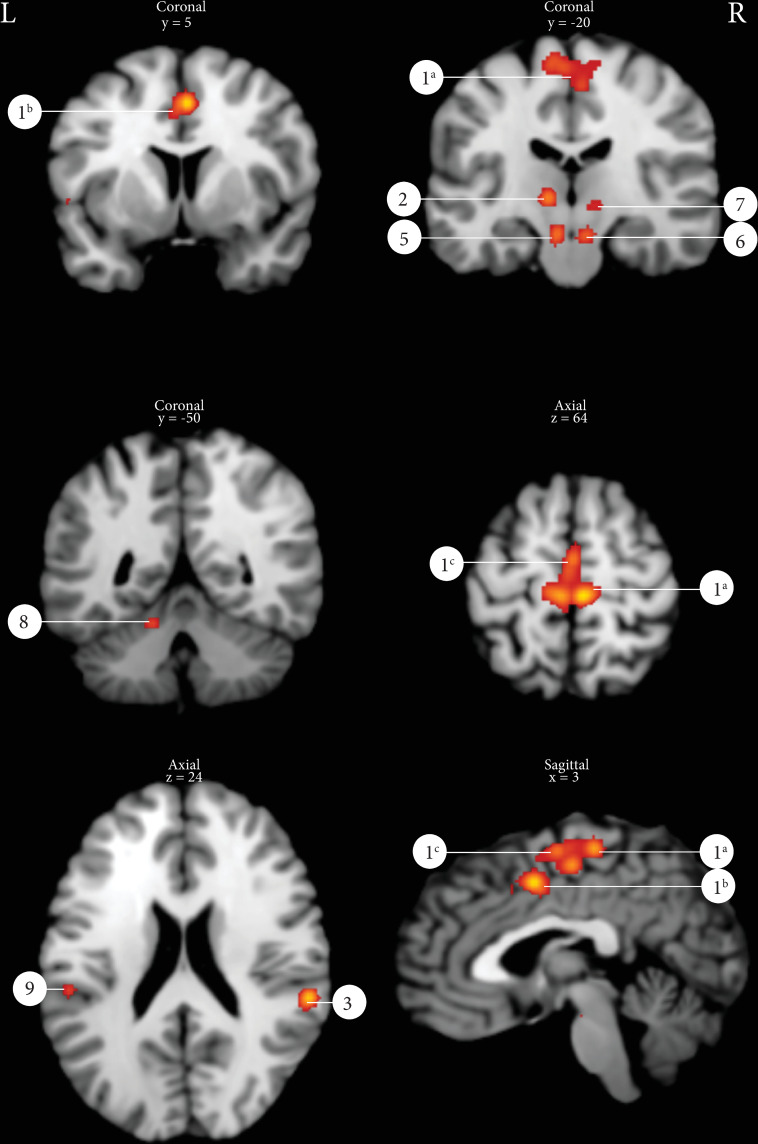
Results of the ALE analysis pelvic floor muscle contraction (p = 0.001 uncorrected with a minimal cluster size of 100m^3^). 1^a^: Primary motor cortex, 1^b^: Mid cingulate gyrus, 1^c^: Supplementary motor area, 2: Thalamus left, 3: Supramarginal gyrus right, 5: Substantia Nigra, 6: Red nucleus, 7: Thalamus right, 8: Cerebellum left, 9: SUpramarginal gyrus left.

The ALE analysis of 133 peak coordinates derived from 12 different studies with a total number of 181 subjects of which 74 men and 95 women (all cluster/peak coordinates summarized in S1 and S2 Tables) yielded 10 active clusters using a statistical threshold of p = 0.001 uncorrected with a minimal cluster size of 100 mm^3^ (Table 4). Fig 2 displays several of the ALE clusters: primary motor cortex, SMA, cingulate gyrus, insula, thalamus, substantia nigra/red nucleus and the cerebellum.

**Table 4:** ALE results of PFMC and micturition tasks.

Cluster	Cluster size	Number of contributing studies	Peaks within cluster	peak coordinates			ALE max. value at peak	Hemisphere R/L	Label
				x	y	z			
1^a^	9552	11 [3,7,10,21–28]	1	6	-26	66	0.0240	R	Primary motor cortex (BA 4)
1^b^			2	2	4	50	0.0213		Mid cingulate gyrus (BA 6)
1^a^			3	-6	-26	66	0.0188	L	Primary motor cortex (Ba 4)
1^c^			4	4	-16	58	0.0182	R	Supplementary motor area (BA 6)
1^c^			5	-2	-14	66	0.0162	L	Supplementary motor area (BA 6)
1^c^			6	0	-8	66	0.0159	L	Supplementary motor area (BA 6)
1^c^			7	-8	-10	74	0.0141	L	Supplementary motor area (BA 6)
1^c^			8	0	14	46	0.0098	L	Supplementary motor area (BA 6)
2	1264	5 [21,24,26–28]	1	-10	-16	4	0.0197	L	Thalamus [BA 50)
3	1024	4 [7,23,26,28]	1	60	-34	22	0.0222	R	Supramarginal gyrus (BA 40)
4	640	3 [7,25,28]	1	58	12	0	0.0151	R	Inferior frontal gyrus, pars opercularis (BA44)
4			2	52	12	-8	0.0105	R	Posterior superior temproral gyrus (BA22)
5	560	3 [24,27,28]	1	-8	-20	-14	0.0151	L	Brainstem, Substania Nigra
6	536	3 [24,27,28]	1	8	-20	-14	0.0140	R	Brainstem, Red Nucleus
7	432	4 [21,24,27,28]	1	12	-18	0	0.0108	R	Thalamus (BA 50)
8	264	2 [21,24]	1	-14	-50	-14	0.0124	L	Cerebellum, Anterior Lobe
9	232	2 [27,28]	1	-56	-30	24	0.0121	L	Supramarginal gyrus (BA 40)
10	128	1 [7]	1	-56	6	2	0.0105	L	Inferior frontal gyrus, pars opercularis (BA44)
1^a^	3336	7 [2,4–6,17,19,20]	1	-6	-24	2	0.0146	L	Thalamus (BA 50)
1^b^			2	8	-32	-22	0.0136		Pontine micturition center
1^c^			3	-2	-30	-8	0.0129		Periaquatuctal grey
1^d^			4	-6	-14	-4	0.0110	L	Thalamus (BA 50)
2	496	2 [4,17]	1	-2	4	44	0.0117		Cingulate Gyrus (BA 6)
3	480	3 [5,17,20]	1	-30	32	36	0.0097	L	Middle Frontal Gyrus (BA 9)
3			2	-30	42	38	0.0093	L	Middle Frontal Gyrus (BA 9)
4	424	2 [17,20]	1	38	34	6	0.0134	R	Insula (BA 13)
5	352	2 [4,20]	1	4	24	48	0.0114		Superior Frontal Gyrus (BA 8)
6	184	2 [17,20]	1	0	-14	-24	0.0096		Ventral pons
7	104	1 [4]	1	-58	6	4	0.0091	L	Inferior frontal gyrus, pars opercularis (BA 44)

**Fig 3:**
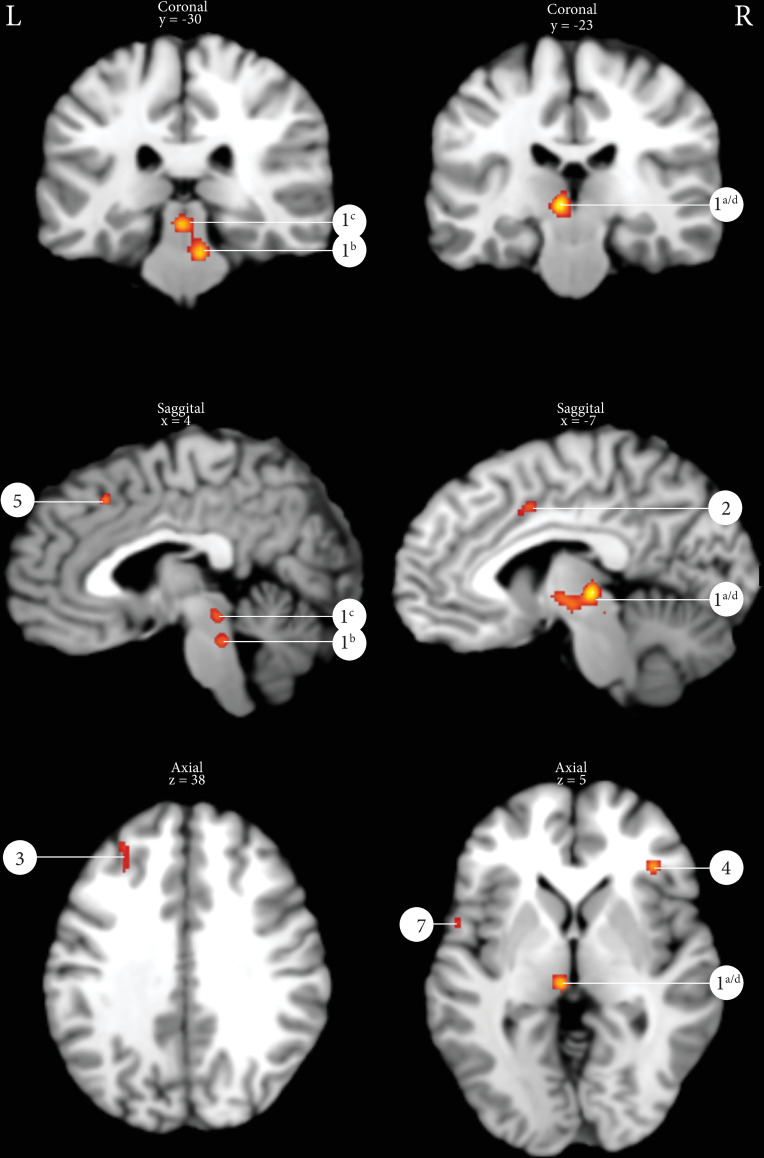
Results of the ALE analysis micturition (p = 0.001 uncorrected with a minimal cluster size of 100m^3^). 1^a/d^: Thalamus, 1^b^: Pontine micturition center, 1^c^: Periaqueductal gray, 2: Cingulate gyrus, 3: Middle frontal gyrus, 4: Insula, 5: Superior frontal gyrus, 6: Ventral pons, 7: Inferior frontal gyrus.

Numbers of clusters correspond with numbers in Figs 2 and 3. All p-values < 0.001. Chosen minimal cluster size: 100 mm^3^.

The ALE analysis delineates where in the brain the convergence across all included imaging studies is higher than it would be expected if results were independently distributed [13]. ALE scores quantify the convergence of foci given the associated spatial uncertainty, while the according p-values quantify, how likely such convergence is under the null-hypothesis of random spatial association [30]. The ALE scores largely depend on the amount of data in the investigated dataset and the applied threshold in each study of the dataset [30]. Since the currently available data are still scarce (PFMC: 12 studies, 181 subjects in total; micturition: 8 studies, 107 subjects in total; recommended: 17 studies with at least 8 subjects in each study) and often represents results with low statistical thresholds (uncorrected p <0.001; recommended: FEW <0.05), the meta-analysis has to be regarded with caution.

#### Micturition

The ALE analyses of 98 peak coordinates derived from 8 different studies with a total number of 107 subjects of which 48 men and 59 women (all peak coordinates summarized in S3 and S4 Tables) yielded 7 active peak activations/clusters using a statistical threshold of p = 0.001 uncorrected with a minimal cluster size of 100 mm^3^ (Table 4). Fig 3 displays exemplarily several of the ALE clusters: PMC, PAG, thalamus, cingulate gyrus, frontal gyrus and insula. Cluster 1 is a merged cluster, showing multiple peak activations in the thalamus, PMC and PAG.

#### Overlap

When the results of the ALE analysis of both PFMC and micturition are displayed on the same MNI brain, two clusters show overlap: the cluster in the mid cingulate gyrus (in both figures cluster #2) and the cluster in the left thalamus (in Fig 2 PFMC cluster #3 and in Fig 3 micturition cluster #1^a/d^). S1 Fig demonstrates the overlap of clusters.

### Risk of bias assessment

**Fig 4:**
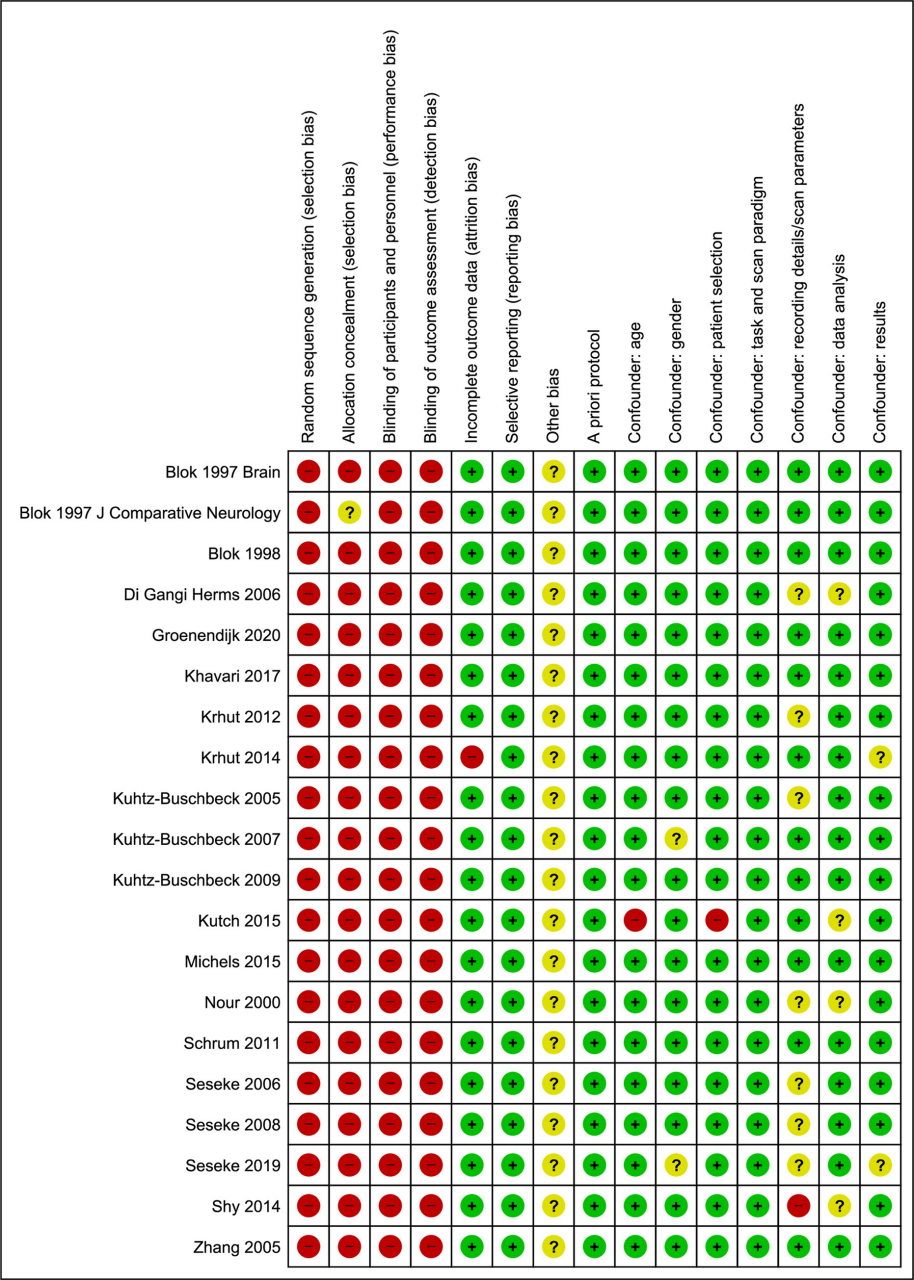
The results of the risk of bias assessment.

The results of the risk of bias assessment by the Cochrane Risk of Bias Assessment Tool with confounding factors are shown in Fig 4.

## Discussion

This systematic review and meta-analysis provide a unique overview on the supraspinal areas involved in LUT motor control (pelvic floor muscle contraction and micturition), showing raw data of the acquired PET and fMRI evidence of the past three decades. Furthermore, ALE-analysis enabled us to extract from all raw data across studies the most commonly and reproducibly activated supraspinal areas involved in LUT motor control.

### Pelvic floor muscle contraction

S1 Table shows that most studies found active clusters in some obvious brain areas involved in pelvic floor motor control, e.g. M1 and SMA. Unexpectedly, three of the studies did not find activation in the M1 [10,21,25] and 5 studies did not find activation in the SMA [3,19,22,23], but all studies found activation in either M1 or SMA. Schrum et al. did not find activation in M1. They used a cytoarchitectonic map [31] to detect that more than 80% of the active cluster found in the medial wall belonged to the SMA. The authors argued that activation in the M1 would have been detected using a more liberal threshold [21]. Several human and non-human primate studies have revealed a somatotopic organization of the SMA [32–34], and showed that the activated areas of the face, the upper limb, and the lower limb were located from anterior to posterior, respectively. This would indicate that activation in the SMA during PFMC might lie in the posterior part of the SMA, which is close to the somatotopic location of activation in the primary motor cortex during PFMC. Furthermore, Di Gangi Herms et al. showed that, after a training interval of 12 weeks regular PFMCs, the number of activated voxels on the SMA decreased significantly compared to before PFMCs training [24]. This demonstrated that the SMA plays an important role in unconditioned motor tasks, like PFMC. Thus, PFMC being an unconditioned task might induce the appearance of large clusters on SMA, and together with its orientation in the posterior SMA, these clusters might be indistinguishable from activation in M1. This could be an explanation for the incongruent findings of the different studies on M1 or SMA.

To our knowledge, our study is the first to confirm the involved brain areas in pelvic floor motor control using an ALE-analysis (Fig 2), namely the: M1, SMA, prefrontal cortex (BA 6 & 9), cingulate gyrus, thalamus, supramarginal gyrus and the anterior lobe of the cerebellum. Putamen activation however, was found in 4 of the included studies [10,17,21,23], but not in our ALE analysis. A possible explanation might be that the coordinates of the peak activations are spread across the putamen and that this did not result in the formation of a cluster in the ALE analysis. The role of the putamen in LUT control has been confirmed earlier, as this structure plays an important role in the corticostriatal pathway which participates in motor action selection and coordination [35].

The ALE analysis revealed two clusters (# 7 and #8) covering the substantia nigra and the red nucleus: parts of the basal ganglia involved in motor tasks [36]. Two studies contributed to the formation of these clusters in the ALE analysis [21,22].

### Micturition

Micturition is a complex process, involving both voluntary and involuntary control regulated by a supraspinal network [11,12]. Until 1996, all available evidence for supraspinal micturition control was obtained from animal studies in cats [37] or from case reports of humans with lesions in the prefrontal cortex [38]. Blok et al. were the first to show brain activation in humans during micturition using PET [2,6]. However, the evidence in humans is still limited, possibly due to the fact that this LUT motor task is complicated to study. This is caused by several reasons; 1) In case of BOLD fMRI, repeated captures of the event-related data are necessary to obtain a higher signal-to-noise ratio, which is hard to establish considering the complex (autonomic and somatic) control of micturition, 2) Involved brain areas lie relatively closely to each other in the brainstem (PMC/PAG/thalamus); distinguishing these areas requires high resolution imaging, 3) A significant number of subjects are not able to void in the scanner, possibly due to the involvement of the prefrontal cortex involved in decision making in social context [1]. The current study describes all PET and fMRI evidence for supraspinal micturition control and included 8 studies with a total of 107 subjects. S3 Table shows all raw data obtained in the last three decades. Using ALE analysis, this study evaluated all evidence and confirmed the involvement of these key brain areas during micturition: the PMC, the PAG, thalamus, cingulate gyrus, prefrontal cortex, the insula and the cerebellum. Cluster 1 is a merged cluster covering the thalamus, PAG, PMC and the cerebellum. It has three obvious peak activations in the thalamus, PAG and PMC as shown in Fig 3. The dorsal pons includes the PMC and the ventral pons includes the pontine continence or storage center [2,6]. These current findings are in line with previous working models on the supraspinal networks supposed to be involved in micturition [1].

S3 and S4 Tables show that all studies found multiple active clusters diffuse across the prefrontal cortex. Peak activations were found in all Brodmann areas within the prefrontal cortex, most of which were located in Brodmann areas 9 and 44. Both areas (the medial prefrontal cortex and the inferior frontal cortex) have been described previously to be involved in micturition [39,40]. The variability of the exact activity location within these areas may result from the variety of different protocols applied in the included studies. The prefrontal cortex has strong connections with other limbic structures–the hypothalamus, amygdala, insula and cingulate gyrus and is known to be involved in the planning of complex cognitive behavior and appropriate social behavior [41]. The voluntary decision when to void is generated in the prefrontal cortex in turn sending efferent signals via the cingulate to the PAG and PMC [1]. The ALE analysis did not show an active cluster in the hypothalamus. However, two of the included studies did find such a cluster as shown in S4 Table [2,6]. As the hypothalamus is part of the limbic system, its role in micturition has been described before [42]. The size of the area is rather small, making detection of active clusters challenging.

### Micturition vs PFMC

This systematic review studies the motor control of the LUT, consisting of one voluntary controlled task (PFMC) and one semi-voluntary controlled task (micturition). Although PFMC and micturition are both motor tasks of the LUT, they have completely different goals. Micturition is a complex coordinated process between the urinary bladder and the external urethral sphincter that aims to empty the bladder periodically, whereas PFMC is a more confined motor task. The latter attribute makes PMFC less complicated to study which may attract more attention and explain the larger number of PFMC studies compared to micturition studies despite it is only a part of the micturition cycle. PFMC concerns the voluntary (M1) contraction on top of the involuntary tonic contraction of the urethral sphincter, which is responsible for urinary continence. The minimal overlap of activated brain areas was therefore expected and demonstrates this distinct working mechanism of the supraspinal organization of both tasks. Comparison of the results shows that only two clusters overlap: midcingulate gyrus (MCG) and the left thalamus. The important role of the MCG in reward-based decision making has been described before [43], and involvement of this area was expected in both LUT motor tasks. The involvement of the basal ganglia was also expected in both tasks. The thalamocortical pathway is important in execution of motor tasks [44]. But, the thalamus is also involved in interoceptive networks, and is therefore important in initiation of micturition [5].

PFMC can be studied during different bladder states (empty vs full), sometimes used to interrupt micturition or suppress the urge to void. In the PFMC ALE, 5 studies investigated PFMC with a full bladder [10,22,23,25,45]. Among those, Zhang et al, compared activation between PFMC with empty vs full bladder and demonstrated differences in SMA, basal ganglia and cerebellum. This might be of great interest towards the clinical applicability of fMRI in patient populations. To better understand the influence of bladder status on pelvic floor control, further studies comparing results at different bladder states are necessary.

Although the current meta-analysis did not specifically focus on sex related differences, data of both men and women were included. Tables 2 and 3 demonstrate that only three studies (2 PFMC and 1 micturition) in this review studied both men and women [4,7,45]. The amount of data in the current systematic review was not sufficient to repeat the ALE analysis separately for men and women to study differences. The studies that included both men and women did compare the results between men and women. Moreover, the study of Seseke et al. focused specifically on this topic in relation to micturition comparing their male results with previous female results [22]. In PFMC, no sex related differences were found [7], however, in another study about external anal sphincter contraction, greater activity was found in men [46]. Various arguments for this difference are described, like a more forceful contraction in men, the obvious anatomical differences between the genitourinary system or a general interhemispheric asymmetry of the human motor cortex related to sexes [7,46,47]. In micturition, stronger task related activity in the right thalamus and other right-hemispherical regions was found in women compared to men [4]. Still, results on this topic are scarce and very heterogeneous. Other studies speculate that women have a stronger brain activity during visceral stimulation than men [48,49].

### Clinical implications & reliability

Compared to the pre-neuroimaging-era, when knowledge of LUT control was mainly based on animal studies which provided a relevant basis to understand particularly spinal and brain stem structures and circuits involved in LUT control [50–52], cortical and subcortical processes in human LUT control were largely unknown. Advances in neuroimaging allowed us to progressively explore this field and to learn which brain areas are involved in human LUT control and how they may be connected. This is of specific value considering that certain LUT diseases such as OAB occur in humans only. In addition, chronic LUT dysfunction may lead to alterations in suprapinal processing of LUT afferent and efferent signaling that itself contribute to or maintain the LUT dysfunction. Therefore, such alterations should be known and understood to improve treatment strategies and to advance the evaluation of treatments targeting suprapinal signal processing such as neuromodulative therapies. This current review and meta-analysis for the first time describes the key brain areas of all previous investigations on LUT motor control, i.e. PFMC and micturition. Knowing the key brain areas and their involvement in different tasks, i.e. PFMC vs micturition, it is important to address new and more specific research questions, to plan according neuroimaging studies, and to address targets for neurofeedback therapy [53]. Specifically interesting in this regard is that our findings clearly demonstrate that the voluntary motor control of PFMC involves typical cortical motor control areas such as M1 and SMA whereas the autonomic detrusor motor control involves brain stem activity, i.e. PAG and PMC, instead. One of the brain areas that is involved in both motor tasks is the cerebellum which seems to play a more relevant role in LUT motor control than previously expected.

Since our knowledge about the physiology of the innervation of the LUT has grown, lately more research is focusing on the results of neuroimaging in patients with LUT disorders, like chronic pelvic pain syndrome [19], stress urinary incontinence (SUI) [24], urge urinary incontinence [54,55] or multiple sclerosis (MS) [26,56]. Khavari et al [56], studied differences in activation after onabotulinumA injections in patients with UUI and MS compared to healthy controls. It was demonstrated that during full urge, patients with UUI and MS show more deactivations compared to healthy controls in cortical and subcortical structures. After onabotulinumA injections, the brain responses were more in agreement with those of healthy controls. Furthermore, a study of Griffiths et al [55]. clearly demonstrated an increased activation in patients with full bladder and UUI compared to healthy controls, particularly in the cingulate gyrus, SMA and prefrontal cortex. What these studies suggest, is that when patients react to therapy, activation patterns had more similarities with those of healthy controls, which is a very usable finding in future implication of fMRI [10,24,26,55–57]. In the current ALE analysis, two studies were included with a patient population instead of healthy controls. One micturition related study included patients with MS [26] and one PFMC related study included patients with SUI [24]. Both ALE analyses (PFMC and micturition) were repeated without inclusion of the patient data to assess the influence on the outcome. Only the amount of clusters changed (the ones with the lowest ALE values disappeared), however, most probably due to the lower number of included subjects in the ALE analysis which is critical for the validity of such analysis and hampers a reasonable interpretation of these differences from a statistical point of view. As visible in the raw data in the supplements, both studies including patient data do not show remarkable outliers, or other distinguishing results compared to the studies including healthy volunteers.

Studying differences in supraspinal activation patterns between healthy controls and patients is important for future clinical implications of neuroimaging and the search towards finding the pathophysiology of diseases within functional urology. The SMA, cingulate gyrus and prefrontal cortex have been independently described as areas of interest when evaluating differences between patients and healthy controls, full or empty bladder and effect of therapies [10,24,26,55–57].

Despite that ALE values cannot be directly compared between different datasets due to the given heterogeneity of the original studies included in the datasets, they still provide a good estimate which brain areas may be more relevant for specific LUT tasks or more consistently activated in states of LUT dysfunction. To allow a meaningful ALE meta-analysis on the differences between healthy controls and patients we would need more original studies that statistically compare both groups, i.e. patients and healthy controls, and provide data (e.g. coordinates) on the group differences that can be included into the meta-analysis. Alternatively, using a rather new approach which aims to compare separate ALE maps of different data sets from healthy controls and patients [58], we would need a larger number of studies investigating healthy controls and patients with a similar total amount of subjects and using same or similar task and imaging protocols. Currently, there are only few functional neuroimaging studies on patients with LUT dysfunction and those studies comparing results to healthy controls do this descriptively only.

The ability of the ALE meta-analysis to elicit relevant brain areas involved in the execution of specific tasks as demonstrated for micturition and PFMT is particularly valuable in the current stage of neuroimaging research in functional urology where mainly whole brain analyses are performed which provide a good overview but may also generate a lot of noise and less relevant secondary activity, specifically when applying lower statistical thresholds (e.g. uncorrected thresholds or voxel-level FDR) to detect any LUT related activity. Here, ALE meta-analysis can help to keep the focus by determining the more consistently involved areas and subsequently also to better specify study hypotheses and designs of future neuroimaging experiments.

Certainly, functional neuroimaging studies on bladder-brain control are a big challenge in many aspects and current study designs and imaging parameters may still be not optimal which may be at least partly reflected by a rather poor reliability [59,60]. Nevertheless, to advance evidence in this field and since validity of ALE analysis depends on the data amount and quality [13,30] more effort is needed to provide such data.

Two aspects appear relevant for this endeavor: a) validation of task and imaging parameters to better understand and control for measurement related bias and to be able to apply higher statistical thresholds. b) correlation of neuroimaging findings to clinical outcomes to better understand the relation between supraspinal activity patterns and dysfunction or symptoms. To better compare between or pool studies, harmonization of protocols (including measures for prevention of movement artefacts, specifically eye movements) and terminology would be of great advantage [61].

### Limitations

The current systematic review and ALE analysis makes all raw data available and creates a clear overview of fMRI and PET imaging of the supraspinal control of the LUT. Our liberal search strategy additionally allowed a very comprehensive review. Nevertheless, there are limitations. This systematic review only included studies using the neuroimaging techniques fMRI or PET, since these techniques yield reliable coordinated-based results, usable for the ALE analysis. Data of other neuroimaging techniques apart from fMRI and PET was therefore not included. Furthermore, to perform a valid ALE analysis and reach sufficient power, the amount of included data is important, which is a criticizable point in the current ALE analysis [30]. However, within the urological field the amount of neuroimaging data is still limited making the use of a liberal threshold inevitable when performing a coordinate based meta-analysis. Altogether, the results of the current systematic review have to be interpreted with the limited amount of data and the risk of bias in mind. Future perspectives are that the preliminary results of the current study encourage to generate more data on this topic, thus allowing for ALE meta-analyses with more significant value. This might enable us to better specify the typical activation patterns (ALE results) in response to certain LUT tasks and to understand if and how pattern may differ between healthy controls and patients with LUT dysfunction.

Another limitation is that this review does not focus on deactivations. As most included studies only report activations, it was decided to focus on activation and not on deactivation. Comparing the overlap of the PFMC and micturition task can therefore be partly biased (see S1 Fig). For example, a cluster that activated during PFMC but deactivated during micturition should be counted as an overlapping cluster, but is not found in the current analysis. Despite both tasks are motor tasks of the LUT, they may be used in opposite direction since the pelvic floor should not be contracted during micturition.

The risk of bias analysis was performed using the Cochrane risk of bias guidelines [16]. As this tool is not specifically designed for neuroimaging studies, the risk of bias might be underestimated [62]. It revealed an unclear risk of bias, which is at least partly related to the great variability of study designs, scan protocols, analysis pathways, and the lack of standardization of reporting methods and outcome measures.

## Conclusions

This systematic review and ALE analysis define all fMRI and PET evidence for the motoric innervation of the LUT. The key brain areas involved in PFMC are M1, SMA, cingulate gyrus, putamen, thalamus, prefrontal cortex, supramarginal gyrus, insula and the cerebellum. The key brain areas involved in micturition are the PAG, PMC, cingulate gyrus, insula, thalamus, prefrontal cortex and the cerebellum. Considering the presented activations, both, PFMC and micturition appear distinct which is in line with their different contextual execution. However, deactivations which are underreported and less well understood could not be systematically considered and may show more overlap than currently presented. Despite that the evidence for neuroimaging of the LUT is still scarce and the affective involvement in performing these tasks makes it challenging to study LUT motor tasks, the involved brain areas in healthy controls seem to be defined, so the step towards defining pathology in a patient population with functional bladder disorders can be made. However, this requires standardization of protocols and task execution.

## Supporting information

S1 Checklist (DOC)

S1 Fig Overlap of tasks. Green: Micturition. Red: Pelvic floor muscle contraction.
(PDF)

S1 Table Peak coordinates of clusters with task specific activity (pelvic floor contraction) in certain brain areas. For purposes of a more concise overview, only brain areas are listed that have been reported to demonstrate task specific activity in at least 3 of the included studies. Further brain areas with task specific activity in individual studies only, are summarized in supplementary 3.
(DOCX)

S2 Table Peak coordinates of clusters with task specific activity (pelvic floor muscle contraction), not shown in supplement 2. (DOCX)

S3 Table Peak coordinates of clusters with task specific activity (micturition) in certain brain areas. For purposes of a more concise overview, only brain areas are listed that have been reported to demonstrate task specific activity in at least 3 of the included studies. Further brain areas with task specific activity in individual studies only, are summarized in supplementary 5.
(DOCX)

S4 Table Peak coordinates of clusters with task specific activity (pelvic floor muscle contraction), not shown in supplement 4. (DOCX)

S1 File Search terms. (EPS)
