# Supplementary figures and images for: A systematic review and activation likelihood estimation meta-analysis of the central innervation of the lower urinary tract: Pelvic floor motor control and micturition
Source: PLoS One. 2021 Feb 3;16(2):e0246042. doi: 10.1371/journal.pone.0246042 (PMC7857581; doi:10.1371/journal.pone.0246042)

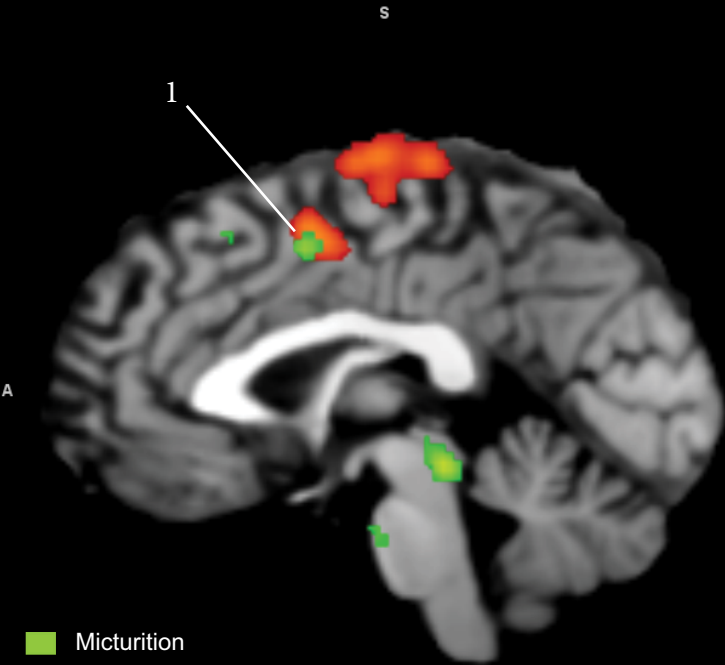

- Micturition
- Pelvic floor muscle contraction

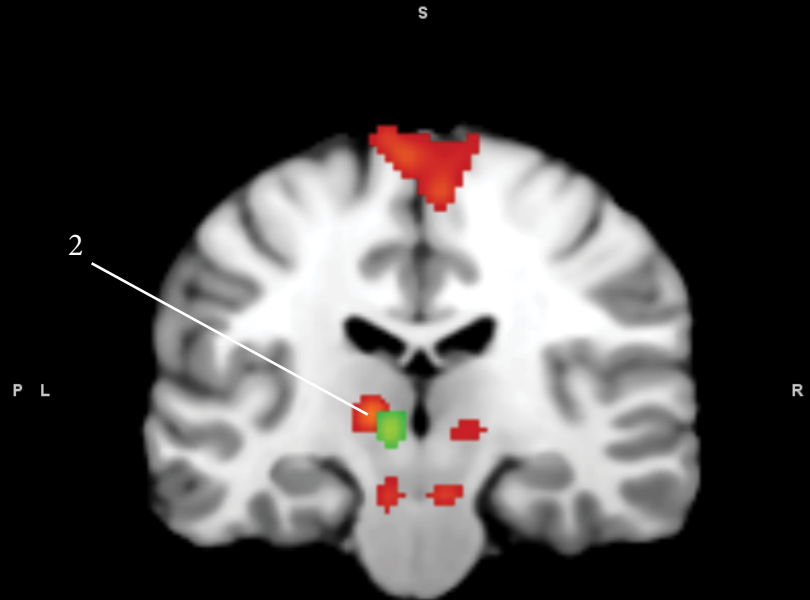

- 1 Overlap cingulate gyrus
- 2 Overlap thalamus

Supplement: S1 Fig — Green: Micturition. Red: Pelvic floor muscle contraction. (PDF) [file pone.0246042.s002.pdf]
