# Supplementary material for: A systematic review and activation likelihood estimation meta-analysis of the central innervation of the lower urinary tract: Pelvic floor motor control and micturition
Source: PLoS One. 2021 Feb 3;16(2):e0246042. doi: 10.1371/journal.pone.0246042 (PMC7857581; doi:10.1371/journal.pone.0246042)
Supplement: S1 Table — For purposes of a more concise overview, only brain areas are listed that have been reported to demonstrate task specific activity in at least 3 of the included studies. Further brain areas with task specific activity in individual studies only, are summarized in supplementary 3. (DOCX) [file pone.0246042.s003.docx]

| Brain area | | M1 | | | | | SMA | | | | | Medial frontal gyrus  (BA 6/9) | | | | | Supramarginal gyrus  (BA 40) | | | | | Cingulate gyrus | | | | |
| --- | --- | --- | --- | --- | --- | --- | --- | --- | --- | --- | --- | --- | --- | --- | --- | --- | --- | --- | --- | --- | --- | --- | --- | --- | --- | --- |
| Orientation | | x | y | z | *T* | *Z* | x | y | Z | *T* | *Z* | x | y | z | *T* | *Z* | x | y | z | *T* | *Z* | x | y | z | *T* | *Z* |
| Groenendijk I, 2020  *MNI* | R | 12 | -24 | 66 | 5.8 |  | 2 | -6 | 64 | 7.6 |  |  |  |  |  |  |  |  |  |  |  | -4 | -6 | 46 | 7.6 |  |
|  | L | -12 | -26 | 66 | 8.7 |  | -2 | -16 | 68 | 7.6 |  |  |  |  |  |  |  |  |  |  |  |  |  |  |  |  |
| Seseke S, 2019  *Talairach* | R | 19 | -30 | 56 | 6.2 |  |  |  |  |  |  | 49 | 5 | 13 | 6.2 |  | 55 | -35 | 26 | 7.7 |  |  |  |  |  |  |
|  | L | -14 | -28 | 61 | 5.9 |  | -2 | -13 | 52 | 7.2 |  | -49 | 4 | 9 | 6.9 |  | -52 | -30 | 23 | 6.2 |  |  |  |  |  |  |
| Kutch JJ, 2015  *MNI* | R | 4 | -26 | 66 |  | 2.3 |  |  |  |  |  |  |  |  |  |  |  |  |  |  |  |  |  |  |  |  |
|  | L |  |  |  |  |  |  |  |  |  |  |  |  |  |  |  |  |  |  |  |  |  |  |  |  |  |
| Kruht J, 2014  *MNI* | R | 4 | -27 | 65 | 10 | 4.5 | 2 | 2 | 50 | 13 | 4.9 | 42 | 36 | 32 |  |  | 62 | -34 | 22 |  |  |  |  |  |  |  |
|  | L | -4 | -27 | 65 | 10 | 4.5 | -2 | 2 | 50 | 13 | 4.9 | -38 | 36 | 32 |  |  |  |  |  |  |  |  |  |  |  |  |
| Schrum A, 2011  *MNI* | R |  |  |  |  |  | 2 | 2 | 62 |  | 6.5 |  |  |  |  |  |  |  |  |  |  | -6 | 6 | 42 |  | 5.8 |
|  | L |  |  |  |  |  |  |  |  |  |  |  |  |  |  |  | -56 | -32 | 22 |  | 5.8 |  |  |  |  |  |
| Seseke S, 2008  *Talairach* | R | 6 | -32 | 60 | 12 |  |  |  |  |  |  |  |  |  |  |  |  |  |  |  |  | 1 | -49 | 31 | 10 |  |
|  | L | -7 | -31 | 59 | 12 |  |  |  |  |  |  | -7 | 50 | 35 | 10 |  |  |  |  |  |  |  |  |  |  |  |
| Kuhtz-Buschbeck, 2007 *MNI* | R |  |  |  |  |  | 0 | -9 | 66 | 9 |  |  |  |  |  |  | 60 | -33 | 21 | 6.2 |  | 0 | 0 | 45 | 7 |  |
|  | L | -9 | -21 | 72 | 6 |  | 0 | -15 | 57 | 9 |  |  |  |  |  |  |  |  |  |  |  |  |  |  |  |  |
| Seseke S, 2006  *Talairach* | R | 1 | -30 | 58 | 10 |  |  |  |  |  |  |  |  |  |  |  |  |  |  |  |  | -7 | -42 | 17 | 6 |  |
|  | L |  |  |  |  |  |  |  |  |  |  |  |  |  |  |  |  |  |  |  |  |  |  |  |  |  |
| Di Gangi Herms A, 2006 *MNI* | R | 15 | -21 | 72 | 6 | 3.8 | 3 | 6 | 51 | 7.5 | 4.1 |  |  |  |  |  |  |  |  |  |  | 0 | 15 | 42 | 5 | 3.4 |
|  | L | -3 | -15 | 66 | 6 | 3.8 |  |  |  |  |  | -9 | -9 | 72 | 4.1 |  |  |  |  |  |  |  |  |  |  |  |
| Kuhtz-Buschbeck, 2005 *MNI* | R |  |  |  |  |  | 6 | -18 | 57 |  | 4.8 |  |  |  |  |  | 57 | -39 | 21 |  | 4.2 | -6 | -6 | 42 |  | 4.3 |
|  | L |  |  |  |  |  |  |  |  |  |  | -9 | -9 | 75 |  | 4 |  |  |  |  |  |  |  |  |  |  |
| Zhang H, 2005  *Talairach* | R |  |  |  |  |  | 8 | -8 | 62 |  | 7.3 |  |  |  |  |  | 64 | -18 | 16 |  | 4.3 |  |  |  |  |  |
|  | L |  |  |  |  |  |  |  |  |  |  |  |  |  |  |  |  |  |  |  |  |  |  |  |  |  |
| Blok B, 1997  *Talairach* | R | 2 | -32 | 66 |  | 3 |  |  |  |  |  | 30 | -20 | 62 |  | 3 |  |  |  |  |  |  |  |  |  |  |
|  | L | -6 | -26 | 74 |  | 3 |  |  |  |  |  |  |  |  |  |  |  |  |  |  |  |  |  |  |  |  |

Supplement 2 shows the peak coordinates of clusters with task specific activity (pelvic floor contraction) in certain brain areas. For purposes of a more concise overview, only brain areas are listed that have been reported to demonstrate task specific activity in at least 3 of the included studies. Further brain areas with task specific activity in individual studies only, are summarized in supplementary 3.

This table continues on the next page.

| Brain area | | Putamen | | | | | Thalamus | | | | | Insula | | | | | Occipital cortex | | | | | Cerebellum | | | | |
| --- | --- | --- | --- | --- | --- | --- | --- | --- | --- | --- | --- | --- | --- | --- | --- | --- | --- | --- | --- | --- | --- | --- | --- | --- | --- | --- |
| Orientation | | x | y | z | *T* | *Z* | x | y | z | *T* | *Z* | x | y | z | *T* | *Z* | x | y | z | *T* | *Z* | x | y | z | *T* | *Z* |
| Groenendijk I, 2020  *MNI* | R | 28 | -4 | 16 | 9.9 |  | 10 | -16 | -14 | 4.1 |  | 46 | 4 | 2 | 6.1 |  |  |  |  |  |  | 10 | -46 | -10 | 3.9 |  |
|  | L | -26 | 0 | 12 | 13 |  | -12 | -16 | 6 | 5.1 |  | -32 | 0 | 12 | 6 |  |  |  |  |  |  | -16 | -48 | -14 | 5.3 |  |
| Seseke S, 2019  *Talairach* | R |  |  |  |  |  | 9 | -13 | 3 | 8.1 |  | 48 | 9 | 7 | 8.7 |  | 0 | -83 | -11 | 4.9 |  | 30 | -51 | -28 | 7.3 |  |
|  | L |  |  |  |  |  | -8 | -15 | 3 | 10.5 |  | -36 | 9 | 7 | 8.1 |  |  |  |  |  |  | -31 | -50 | -28 | 7.8 |  |
| Kutch JJ, 2015  *MNI* | R |  |  |  |  |  |  |  |  |  |  |  |  |  |  |  |  |  |  |  |  |  |  |  |  |  |
|  | L |  |  |  |  |  |  |  |  |  |  |  |  |  |  |  |  |  |  |  |  |  |  |  |  |  |
| Kruht J, 2014  *MNI* | R |  |  |  |  |  |  |  |  |  |  |  |  |  |  |  |  |  |  |  |  |  |  |  |  |  |
|  | L |  |  |  |  |  |  |  |  |  |  |  |  |  |  |  |  |  |  |  |  |  |  |  |  |  |
| Schrum A, 2011 *MNI* | R | 34 | 2 | 4 |  | 6.3 | 8 | -22 | -2 |  | 6.1 | 34 | 18 | 2 |  | 6.2 |  |  |  |  |  |  |  |  |  |  |
|  | L | -26 | -14 | 4 |  | 5.8 | -10 | -18 | 4 |  | 5.7 | -44 | 0 | -4 |  | 7 |  |  |  |  |  | -4 | -58 | -32 |  | 5 |
| Seseke S, 2008  *Talairach* | R |  |  |  |  |  | 13 | -20 | 3 | 7.1 |  |  |  |  |  |  | 24 | -83 | 11 | 9.2 |  | 19 | -54 | -17 | 10 |  |
|  | L |  |  |  |  |  | -14 | -22 | 3 | 6.8 |  |  |  |  |  |  | -16 | -81 | 15 | 9.6 |  | -14 | -49 | -14 | 9.4 |  |
| Kuhtz-Buschbeck, 2007 *MNI* | R |  |  |  |  |  |  |  |  |  |  | 51 | 12 | -9 | 6.6 |  |  |  |  |  |  | 6 | -42 | -24 |  |  |
|  | L |  |  |  |  |  |  |  |  |  |  |  |  |  |  |  |  |  |  |  |  |  |  |  |  |  |
| Seseke S, 2006  *Talairach* | R | 30 | -18 | 16 | 6 |  |  |  |  |  |  |  |  |  |  |  | 40 | -70 | 28 | 5.5 |  |  |  |  |  |  |
|  | L | -29 | -18 | 12 | 6 |  |  |  |  |  |  |  |  |  |  |  | -36 | -69 | 25 | 7.1 |  | -1 | -47 | -14 |  |  |
| Di Gangi Herms A, 2006 *MNI* | R |  |  |  |  |  |  |  |  |  |  | 57 | 15 | -3 | 7.3 | 4.1 |  |  |  |  |  | 0 | -75 | -12 | 4.6 | 3.2 |
|  | L |  |  |  |  |  |  |  |  |  |  |  |  |  |  |  |  |  |  |  |  | -36 | -60 | -21 | 4.4 | 4.1 |
| Kuhtz-Buschbeck, 2005 *MNI* | R |  |  |  |  |  |  |  |  |  |  | 45 | 9 | -9 |  | 4.4 |  |  |  |  |  |  |  |  |  |  |
|  | L |  |  |  |  |  | -12 | -12 | 9 |  | 3.4 |  |  |  |  |  |  |  |  |  |  |  |  |  |  |  |
| Zhang H, 2005  *Talairach* | R | 28 | 6 | -2 |  | 4.5 |  |  |  |  |  |  |  |  |  |  |  |  |  |  |  | 18 | -32 | -10 |  | 4.2 |
|  | L | -24 | 10 | -6 |  | 4 |  |  |  |  |  |  |  |  |  |  |  |  |  |  |  |  |  |  |  |  |
| Blok B, 1997  *Talairach* | R |  |  |  |  |  |  |  |  |  |  |  |  |  |  |  |  |  |  |  |  |  |  |  |  |  |
|  | L |  |  |  |  |  | -10 | -4 | 4 |  | 3.2 |  |  |  |  |  |  |  |  |  |  | -6 | -52 | -12 |  | 3.9 |
