# Supplementary material for: A systematic review and activation likelihood estimation meta-analysis of the central innervation of the lower urinary tract: Pelvic floor motor control and micturition
Source: PLoS One. 2021 Feb 3;16(2):e0246042. doi: 10.1371/journal.pone.0246042 (PMC7857581; doi:10.1371/journal.pone.0246042)
Supplement: S2 Table — (DOCX) [file pone.0246042.s004.docx]

|  |  |  | **x** | **y** | **z** | ***T*** | ***Z*** | **Hemisphere** | **Area** |  |  | | | |
| --- | --- | --- | --- | --- | --- | --- | --- | --- | --- | --- | --- | --- | --- | --- |
| Groenendijk, I.M. | 2020 | MNI | -48 | -2 | 52 | 3,3 |  | L | inferolateral M1 | |  | | | |
|  |  |  | 42 | -2 | 54 | 3,7 |  | R | inferolateral M1 | |  | | | |
| Seseke, S. | 2019 | TAL | -1 | -25 | -36 | 5.1 |  |  | Pontine micturition center | | | | | |
|  |  |  | 7 | -23 | -8 | 7.1 |  | R | Periaqueductal gray | | | | | |
|  |  |  | -7 | -21 | -8 | 7.3 |  | L | Periaqueductal gray | | | | | |
|  |  |  | 20 | -6 | 0 | 6.5 |  | R | Globus pallidus | | | | | |
|  |  |  | -25 | -8 | 0 | 6.7 |  | L | Globus pallidus | | | | | |
|  |  |  | -1 | 7 | 49 | 9.1 |  |  | Pre-SMA | | | | | |
| Kutch, J.J. | 2015 | MNI | All clusters presented in table | | | 3 |  |  |  |  |  | | | |
| Kruht, J. | 2014 | MNI | 43 | -13 | 63 | 8,2 | 3,6 | L | precentral gyrus | |  | | | |
| Schrum, A. | 2011 | MNI | -52 | -40 | 48 |  | 5,3 | L | posterior parietal cortex | | | | | |
|  |  |  | 50 | -30 | 16 |  | 6,6 | R | patietal operculum | |  | | | |
|  |  |  | -8 | -20 | -18 |  | 5,8 | L | upper ventral pons | |  | | | |
|  |  |  | 4 | -20 | -16 |  | 5,3 | R | upper ventral pons | |  | | | |
| Seseke, S. | 2008 | TAL | -42 | -59 | 15 | 9,9 |  | L | middle temporal cortex | | | | | |
|  |  |  | 2 | -49 | -18 | 9,1 |  | R | vermis |  |  | | | |
|  |  |  | 4 | -19 | -30 | 6,2 |  | R | ventral pons | |  | | | |
|  |  |  | 8 | -18 | -11 | 4,6 |  | R | peri-aquaductal grey | | | | | |
|  |  |  | -7 | -17 | -9 | 4,6 |  | L | peri-aquaductal grey | | | | | |
| Kuhtz-Buschbeck, J.P. | 2007 | MNI | 3 | 3 | 48 | 7,5 |  | R | superior frontal gyrus | | | | | |
|  |  |  | -57 | 6 | 0 | 8,7 |  | L | frontal operculum | |  | | | |
|  |  |  | 60 | 12 | 0 | 9,2 |  | R | frontal operculum | |  | | | |
| Seseke, S. | 2006 | TAL | All clusters presented in table 3 | | | |  |  |  | |  | | | |
| Di Gangi Herms, A.M.R | 2006 | MNI | 21 | -30 | 69 | 5,2 | 3,5 | R | superior lateral postcentral gyrus | | | | | |
|  |  |  | -18 | -33 | 72 | 4,7 | 3,3 | L | superior lateral postcentral gyrus | | | | | |
|  |  |  | -54 | 3 | 42 | 8,56 | 4,4 | L | premotor area | |  |  | | |
| Kuhtz-Buschbeck | 2005 | MNI | All clusters presented in table | | | 3 |  |  |  |  |  |  | | |
| Zhang, H. 2005 | 2005 | TAL | -2 | -30 | 70 |  | 5,6 | L | paracentral lobule | |  |  | | |
|  |  |  | 4 | -78 | 44 |  | 5,3 | R | percuneus | |  |  | | |
|  |  |  | 18 | -32 | -10 |  | 4,2 | R | parahippocampal gyrus/limbic lobe | | | | | |
|  |  |  | -20 | 18 | 6 |  | 3,9 | L | putamen |  |  | | |  |
|  |  |  | 10 | -38 | 68 |  | 3,9 | R | paracentral lobule | |  | | |  |
| Blok, B.F.M. | 1997 | TAL | 16 | 30 | 54 |  | 3,7 | R | superior frontal gyrus | | | | |  |
|  |  |  | 24 | 16 | 32 |  | 3,7 | R | medial frontal gyrus | | | |  |  |
|  |  |  | 36 | -52 | 20 |  | 3,1 | R | temporal lobe | | | |  |  |
|  |  |  | 16 | 50 | 14 |  | 2,8 | R | anterior cingulate and medial frontal gyrus | | | | | |
