# Supplementary material for: A systematic review and activation likelihood estimation meta-analysis of the central innervation of the lower urinary tract: Pelvic floor motor control and micturition
Source: PLoS One. 2021 Feb 3;16(2):e0246042. doi: 10.1371/journal.pone.0246042 (PMC7857581; doi:10.1371/journal.pone.0246042)
Supplement: S3 Table — For purposes of a more concise overview, only brain areas are listed that have been reported to demonstrate task specific activity in at least 3 of the included studies. Further brain areas with task specific activity in individual studies only, are summarized in supplementary 5. (DOCX) [file pone.0246042.s005.docx]

| Brain area | | Periaquaductal grey | | | | | Pons (pontine micturition center) | | | | | Cingulate gyrus | | | | | Insula | | | | | Thalamus | | | | |
| --- | --- | --- | --- | --- | --- | --- | --- | --- | --- | --- | --- | --- | --- | --- | --- | --- | --- | --- | --- | --- | --- | --- | --- | --- | --- | --- |
| Orientation | | x | y | z | *T* | *Z* | x | y | z | *T* | *Z* | x | y | z | *T* | *Z* | x | y | z | *T* | *Z* | x | y | z | *T* | *Z* |
| Khavari R, 2017  *MNI* | R | 8 | -27 | -17 | -3.4 |  | 0 | -13 | -23 | -4.4 |  | -6 | 7 | 44 | -4.8 |  |  |  |  |  |  |  |  |  |  |  |
|  | L |  |  |  |  |  |  |  |  |  |  |  |  |  |  |  | -45 | -3 | 3 | -4.8 |  | -6 | -24 | 1 | -4.5 |  |
| Michels L, 2015  *MNI* | R | -6 | -15 | -6 | 2.9 |  | 6 | -30 | -24 | 5.5 |  | 12 | -30 | 42 | 5.5 |  | 36 | 18 | 9 | 3.3 |  |  |  |  |  |  |
|  | L |  |  |  |  |  |  |  |  |  |  |  |  |  |  |  | -33 | 24 | 3 | 3 |  | -18 | -18 | 6 | 4.8 |  |
| Sky M, 2014  *MNI* | R |  |  |  |  |  | 0 | -16 | -30 | 5.8 |  | -7 | 14 | 39 | 5.8 |  |  |  |  |  |  |  |  |  |  |  |
|  | L |  |  |  |  |  |  |  |  |  |  |  |  |  |  |  |  |  |  |  |  | -9 | -22 | 0 | 5.8 |  |
| Kruht J, 2012  *MNI* | R |  |  |  |  |  |  |  |  |  |  | -10 | 37 | -6 | 3.6 | 2.4 |  |  |  |  |  |  |  |  |  |  |
|  | L |  |  |  |  |  |  |  |  |  |  |  |  |  |  |  |  |  |  |  |  |  |  |  |  |  |
| Kuhtz-Buschbeck, 2009 *MNI* | R | -3 | -30 | -9 | 2.4 | 2.3 |  |  |  |  |  | 0 | 3 | 45 | 9 | 6.3 | 45 | 15 | -9 | 7.3 | 5.6 |  |  |  |  |  |
|  | L |  |  |  |  |  |  |  |  |  |  |  |  |  |  |  | -42 | 12 | -3 | 6.7 | 5.2 |  |  |  |  |  |
| Nour, S 2000  *Talairach* | R | 4 | -26 | -6 |  | 5.2 |  |  |  |  |  | -4 | 0 | 46 |  | 5 | 36 | 4 | 14 |  | 6.2 |  |  |  |  |  |
|  | L |  |  |  |  |  |  |  |  |  |  |  |  |  |  |  |  |  |  |  |  | -4 | -24 | 6 |  | 4.9 |
| Blok B, 1998  *Talairach* | R | 2 | -38 | -24 |  | 2.8 | 12 | -36 | -28 |  | 3.6 |  |  |  |  |  |  |  |  |  |  |  |  |  |  |  |
|  | L |  |  |  |  |  |  |  |  |  |  |  |  |  |  |  |  |  |  |  |  |  |  |  |  |  |
| Blok B, 1997  *Talairach* | R | 4 | -34 | -12 |  | 3.1 | 10 | -42 | -24 |  | 2.6 |  |  |  |  |  |  |  |  |  |  |  |  |  |  |  |
|  | L |  |  |  |  |  |  |  |  |  |  |  |  |  |  |  |  |  |  |  |  |  |  |  |  |  |

Supplement 4 shows the peak coordinates of clusters with task specific activity (micturition) in certain brain areas. For purposes of a more concise overview, only brain areas are listed that have been reported to demonstrate task specific activity in at least 3 of the included studies. Further brain areas with task specific activity in individual studies only, are summarized in supplementary 5.

This table continues on the next page

| Brain area | | Inferior frontal gyrus  (BA 11, 44-47) | | | | | Mid frontal gyrus  (BA 9 or 10) | | | | | Cerebellum | | | | |
| --- | --- | --- | --- | --- | --- | --- | --- | --- | --- | --- | --- | --- | --- | --- | --- | --- |
| Orientation | | x | y | z | *T* | *Z* | x | y | z | *T* | *Z* | x | y | z | *T* | *Z* |
| Khavari R, 2017  *MNI* | R | 38 | 33 | 6 | -5.2 |  |  |  |  |  |  | 7 | -61 | -52 | -3.4 |  |
|  | L | -39 | 34 | 5 | -3.9 |  | -31 | 44 | 39 | -3.7 |  |  |  |  |  |  |
| Michels L, 2015  *MNI* | R | 48 | 45 | -6 | 7.3 |  |  |  |  |  |  | 30 | -57 | -45 | 3.5 |  |
|  | L | -57 | 6 | 14 | 4.8 |  | -30 | 30 | 36 | 5.3 |  | -42 | -51 | -39 | 5.2 |  |
| Sky M, 2014  *MNI* | R | 40 | 35 | 6 | 5.8 |  |  |  |  |  |  |  |  |  |  |  |
|  | L | -47 | 27 | -4 | 5.8 |  | -28 | 37 | 36 | 5.8 |  | -35 | -32 | -32 | 5.8 |  |
| Kruht J, 2012  *MNI* | R | 11 | 42 | -12 | 3.6 | 2.4 |  |  |  |  |  |  |  |  |  |  |
|  | L | -11 | 43 | -12 | 3.6 | 2.4 |  |  |  |  |  |  |  |  |  |  |
| Kuhtz-Buschbeck, 2009 *MNI* | R | 60 | 12 | 6 | 6.7 | 5.2 | 48 | 51 | 6 | 7.5 | 5.6 |  |  |  |  |  |
|  | L | -57 | 6 | 3 | 8.1 | 5.9 |  |  |  |  |  |  |  |  |  |  |
| Nour, S 2000  *Talairach* | R | 48 | 4 | 10 |  | 5.7 |  |  |  |  |  | 30 | -42 | -50 |  | 5 |
|  | L | -66 | 4 | 14 |  | 5.3 |  |  |  |  |  | -38 | -54 | -34 |  | 6 |
| Blok B, 1998  *Talairach* | R | 52 | 24 | 12 |  | 3.1 |  |  |  |  |  |  |  |  |  |  |
|  | L |  |  |  |  |  |  |  |  |  |  |  |  |  |  |  |
| Blok B, 1997  *Talairach* | R | 48 | 26 | -4 |  | 4.6 |  |  |  |  |  |  |  |  |  |  |
|  | L |  |  |  |  |  |  |  |  |  |  |  |  |  |  |  |
