# Supplementary material for: A systematic review and activation likelihood estimation meta-analysis of the central innervation of the lower urinary tract: Pelvic floor motor control and micturition
Source: PLoS One. 2021 Feb 3;16(2):e0246042. doi: 10.1371/journal.pone.0246042 (PMC7857581; doi:10.1371/journal.pone.0246042)
Supplement: S4 Table — (DOCX) [file pone.0246042.s006.docx]

|  |  |  | **x** | **y** | **z** | ***T*** | ***Z*** | **Hemisphere** | **brain area** |
| --- | --- | --- | --- | --- | --- | --- | --- | --- | --- |
| Khavari, R. | 2017 | MNI | -6 | -13 | 15 | -3,8 |  | L | thalamus |
|  |  |  | 0 | -29 | 26 | -3,6 |  |  | cingulate gyrus |
| Michels, L. | 2015 | MNI | -57 | -27 | 18 | 6,7 |  | L | postcentral gyrus |
|  |  |  | 63 | 18 | 18 | 3,4 |  | R | inferior frontal gyrus |
|  |  |  | -51 | -63 | 3 | 5,4 |  | L | middle temporal gyrus |
|  |  |  | 62 | -18 | 18 | 7,2 |  | R | postcentral gyrus |
|  |  |  | 15 | -72 | 48 | 4,6 |  | R | percuneus |
| Shy, M. | 2014 | MNI | -44 | 1 | 28 | 5,7 |  | L | precentral gyrus |
|  |  |  | -19 | -9 | 52 | 5,7 |  | L | middle frontal gyrus |
|  |  |  | -16 | 17 | 37 | 5,7 |  |  | cingulate gyrus |
|  |  |  | 21 | 10 | -14 | 5,7 |  | R | subcalossal gyrus |
|  |  |  | 8 | 22 | 44 | 5,7 |  | R | cingulate gyrus |
|  |  |  | -33 | 27 | 30 | 5,7 |  | L | middle frontal gyrus |
|  |  |  | -5 | 21 | 47 | 5,7 |  | L | medial frontal gyrus |
|  |  |  | -18 | 28 | 52 | 5,7 |  | L | superior frontal gyrus |
|  |  |  | -32 | -11 | 47 | 5,7 |  | L | middle frontal gyrus |
|  |  |  | -42 | 33 | 26 | 5,7 |  | L | middle frontal gyrus |
|  |  |  | -32 | 12 | 33 | 5,7 |  | L | middle frontal gyrus |
| Krhut, J. | 2012 | MNI | -16 | -39 | -7 | 4,9 | 2,9 | L | parahippocampal gyrus |
|  |  |  | 10 | 37 | -6 | 3,6 | 2,4 | R | cingulate gyrus |
|  |  |  | -56 | -50 | -10 | 4,9 | 2,9 | L | temporal gyrus/inferior temporal gyrus |
|  |  |  | 53 | -24 | 28 | 5 | 3,2 | R | supramarginal gyrus |
| Kuhtz-Buschbeck, J. | 2009 | MNI | 0 | -3 | 57 | 7,7 | 5,7 | L | supplementary motor area |
|  |  |  | 3 | -12 | 63 | 7,4 | 5,6 | R | supplementary motor area |
|  |  |  | 36 | 57 | 15 | 7 | 5,4 | R | middle frontal gyrus |
|  |  |  | 36 | 51 | 24 | 6,6 | 5,2 | R | middle frontal gyrus |
|  |  |  | 57 | 9 | 9 | 6,4 | 5,1 | R | opercullum |
|  |  |  | 48 | -57 | 51 | 6,3 | 5 | R | inferior parietal lobe |
|  |  |  | 6 | 0 | 72 | 6,2 | 5 | R | supplementary motor area |
|  |  |  | 63 | -51 | 33 | 6,2 | 5 | R | angular gyrus |
|  |  |  | 3 | 24 | 48 | 6,1 | 4,9 | R | superior frontal gyrus |
| Nour, S. | 2000 | Talairach | -24 | -66 | -48 |  | 5,1 | L | cerebellum |
|  |  |  | 2 | -56 | -24 |  | 5,6 | R | cerebellum |
|  |  |  | 2 | -44 | -8 |  | 5,2 | R | cerebellum |
|  |  |  | -6 | -10 | -4 |  | 4,8 | L | anterior mesencephalon |
|  |  |  | 16 | -4 | 0 |  | 5,4 | R | globus pallidus |
|  |  |  | -16 | -8 | 2 |  | 4,7 | L | globus pallidus |
|  |  |  | 28 | 24 | 4 |  | 4,7 | R | insula |
|  |  |  | 36 | -14 | 8 |  | 4,8 | R | insula |
|  |  |  | -22 | -8 | 72 |  | 5 | L | superior frontal gyrus |
|  |  |  | -6 | -18 | 74 |  | 5,1 | L | supplementary motor area |
|  |  |  | -10 | -40 | 76 |  | 5,1 | L | post and precentral gyrus |
|  |  |  | 20 | -44 | 74 |  | 5,5 | R | postcentral gyrus |
|  |  |  | 56 | -30 | 24 |  | 5,1 | R | supramarginal gyrus |
| Blok. B. | 1998 | Talairach | -8 | 10 | -8 |  | 2,6 | L | hypothalamus |
| Blok. B. | 1997 | Talairach | 50 | -2 | -12 |  | 3,1 | R | medial temporal gyrus |
|  |  |  | -4 | -4 | -4 |  | 3,4 | L | hypothalamus |
